# Clinical relevance of hyperamylasemia and pancreatitis-like imaging linked with accidental hypothermia

**DOI:** 10.1371/journal.pone.0353128

**Published:** 2026-07-07

**Authors:** Erika Yawata, Yuuki Bamba, Yosuke Kawai, Hiroyuki Honda, Kei Nishiyama

**Affiliations:** Department of Advanced Critical Care and Emergency Center, Niigata University Medical and Dental Hospital, Niigata City, Niigata Prefecture, Japan; Tottori University Faculty of Medicine Graduate School of Medicine: Tottori Daigaku Igakubu Daigakuin Igakukei Kenkyuka, JAPAN

## Abstract

**Background:**

Elevated pancreatic enzymes and pancreatitis-like imaging findings have been reported in patients with accidental hypothermia; however, their clinical significance remains unclear. This study aimed to explore a serum amylase threshold that may support consideration of computed tomography (CT) for evaluating pancreatitis-like findings in accidental hypothermia and describe the clinical course of affected patients.

**Methods:**

We conducted a retrospective single-center observational study of adult patients with accidental hypothermia admitted to a tertiary emergency and critical care center in Japan between November 2011 and April 2023. Accidental hypothermia was defined as a core body temperature <35°C. Receiver operating characteristic (ROC) analysis was performed to evaluate the ability of serum amylase levels to identify pancreatitis-like CT findings. Patients with hyperamylasemia and pancreatitis-like CT findings were descriptively analyzed.

**Results:**

Among 169 patients included in the study, 36 (21.3%) had hyperamylasemia. Pancreatitis-like CT findings were observed in 14 patients, of whom 13 had hyperamylasemia. ROC analysis among patients who underwent CT evaluation identified 428 IU/L as a serum amylase threshold associated with pancreatitis-like CT findings (area under the curve, 0.91; sensitivity, 93%; specificity, 86%). The positive and negative predictive values were 44.8% and 99.0%, respectively. Most CT abnormalities consisted of localized peripancreatic fat stranding, fluid collection, or pancreatic enlargement. No patients developed pancreatic necrosis or required invasive pancreatic intervention. Most patients were managed conservatively with fluids and nutritional support, and short-term outcomes were generally favorable.

**Conclusions:**

In patients with accidental hypothermia, serum amylase levels ≥428 IU/L may support consideration of CT evaluation of pancreatitis-like findings. Although hyperamylasemia alone showed limited positive predictive value, most patients with pancreatitis-like CT findings had favorable short-term outcomes with conservative management.

## Introduction

Acute pancreatitis is an inflammatory disease that can present with a variety of symptoms, ranging from mild localized inflammation to severe necrotizing pancreatitis with organ failure. The mortality rate for acute pancreatitis is around 2%, but in severe cases involving organ damage, it can reach 30% [1]. The diagnosis of acute pancreatitis requires the presence of at least two of the following three criteria (1) Abdominal pain consistent with acute pancreatitis (2) Serum lipase or amylase activity three times the normal level (3) Contrast-enhanced computed tomography (CT) or magnetic resonance imaging [2].

Accidental hypothermia is defined as a condition in which the body temperature drops below 35°C (95°F). This condition occurs when the body is exposed to an excessively cold external environment or when the body temperature control mechanism malfunctions [3]. Elevated serum amylase levels and CT findings suggestive of pancreatitis have been reported in patients with accidental hypothermia [4]. There have also been reports of cases where fatal accidental hypothermia was complicated by acute pancreatitis [5]. It has been suggested that an initial core body temperature of 28.5°C or below may lead to the development of acute pancreatitis [6].

However, the mechanisms underlying hyperamylasemia and pancreatitis-like imaging findings in accidental hypothermia remain unclear. It is hypothesized that circulatory failure is involved in the development of pancreatitis with hypothermia, unlike general cases of acute pancreatitis due to gallstones or alcohol [7,8]. Data remain limited regarding whether pancreatitis-like findings associated with accidental hypothermia follow a clinical course similar to that of typical acute pancreatitis.

This study aimed to explore a serum amylase threshold that may support consideration of CT evaluation of pancreatitis-like findings in patients with accidental hypothermia. We also investigated the imaging findings, management, and short-term outcomes of patients with hyperamylasemia and pancreatitis-like CT findings.

## Methods

### Study design and setting, and ethics

A retrospective single-center observational study was conducted at the Niigata University Medical and Dental Hospital in Japan, which is a tertiary medical center that covers a population of approximately 1 million people.

This study complied with the principles of the Declaration of Helsinki and the ethical guidelines. The study protocol was approved by the ethics committee of Niigata University Medical and Dental Hospital (#2023−0229). This research does not involve any new invasion of the patient, so　the requirement for written informed consent was waived. The purpose of the study and opt-out opportunities were provided on the hospital’s website.

### Population and study protocol

The study population consisted of patients who were transported to the emergency room and were admitted to the Intensive Care Unit (ICU) or the critical care center between November 2011 and April 2023 with accidental hypothermia. We included patients aged 18 years or older. Accidental hypothermia was defined as a core temperature, bladder temperature, or rectal temperature of less than 35°C (95°F).

The data taken from the medical records included age, gender, initial core temperature, administration of catecholamine, type of catecholamine, use of a ventilator, renal replacement therapy, ICU stay or emergency critical care unit stay, death during the ICU or emergency critical care unit stay, place of discharge, length of stay, peak serum amylase levels up to first three times after admission, whether or not a CT scan was performed, and pancreatitis-like CT findings. CT examinations were generally performed during the initial emergency department evaluation to investigate the cause of hypothermia.

The evidence of suspected pancreatitis on CT were determined based on the reports of the findings of emergency department physicians or gastroenterologists, and reports of radiologists. Serum amylase was measured using the Et-G7PNP enzymatic method as part of routine laboratory evaluation for organ dysfunction at the time of emergency department presentation and during hospitalization.　Hyperamylasemia was defined as a serum amylase level of 398 IU/L or higher, according to the revised Atlanta classification of acute pancreatitis [2]. Serum lipase was not available for rapid measurement in our institution during the study period.

The outcome was the diagnostic performance of serum amylase levels for identifying pancreatitis-like CT findings. Patients with pancreatitis-like findings on CT were descriptively investigated in the group of patients with high amylase levels.

### Statistical analysis

The Wilcoxon rank sum test was used to compare the hyperamylasemia group with the no hyperamylasemia group with respect to age, initial core temperature, peak of serum amylase, length of ICU stays or emergency critical care unit stay, and length of hospital stay. Fisher’s exact test was performed for sex, findings of acute pancreatitis on CT, administration of catecholamine, use of ventilation, renal replacement therapy, ICU mortality, or emergency critical care unit.

**Fig 1:**
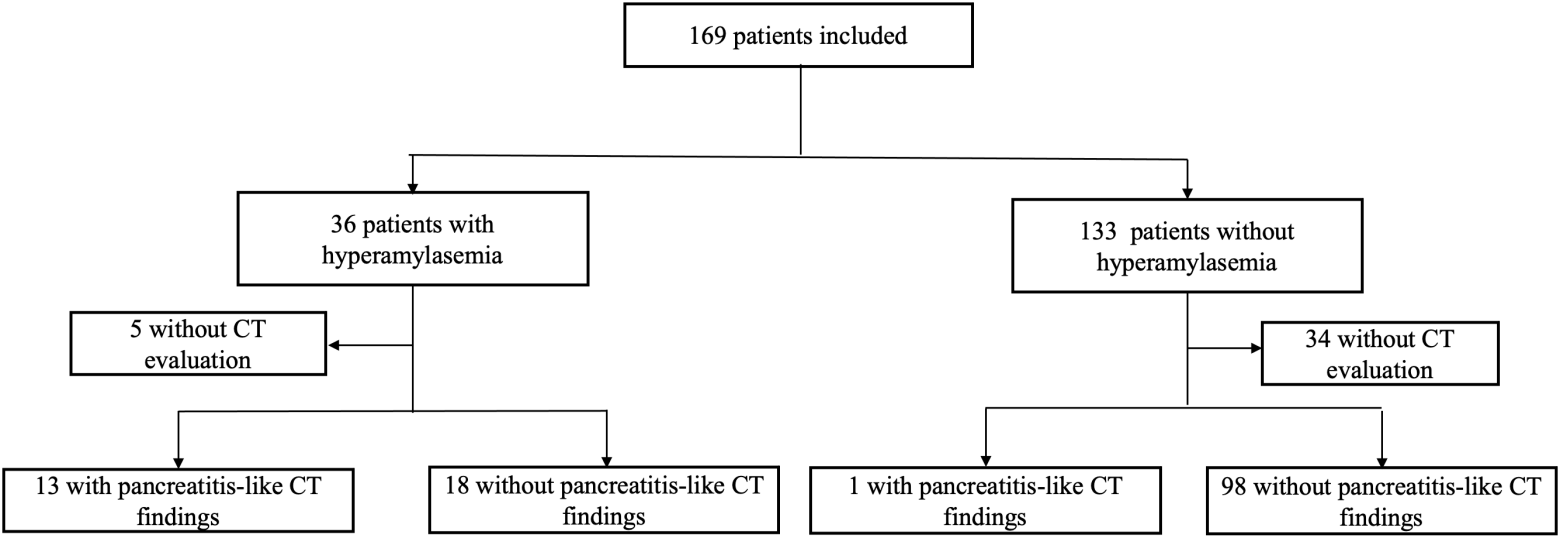
Flow chart of the study. Study flow diagram illustrating the distribution of patients with and without hyperamylasemia, the use of computed tomography (CT), and CT findings suggestive of acute pancreatitis among patients with accidental hypothermia.

Receiver operating characteristic (ROC) curve analysis was performed to evaluate the ability of serum amylase levels to discriminate pancreatitis-like CT findings. Positive predictive value (PPV)and negative predictive value (NPV) were also calculated. Diagnostic accuracy measures, including sensitivity, specificity, PPV, and NPV, were calculated among patients who underwent CT evaluation. Patients who did not undergo CT are shown in the patient flow diagram (Fig 1) but were not included in the diagnostic accuracy analysis.

Additionally, missing values were excluded. A value of *p* < 0.05 was considered statistically significant. We used JMP Pro 16 (SAS Institute Inc., NC, USA) for data analysis.

## Results

In 169 cases, 36 patients had hyperamylasemia, and 13 of them had pancreatitis-like CT findings. CT scans were performed on 99 patients in no hyperamylasemia group, and only one patient had radiological findings of pancreatitis (Fig 1).

Patient characteristics are summarized in Table 1. The hyperamylasemia group had significantly higher amylase levels 714 IU/L (IQR, 551–1091), compared to the no hyperamylasemia group 95 IU/L (IQR,63–183). There was no difference in initial core temperature and use of vasopressors between the two groups. Deaths during ICU or emergency critical care unit stay were 6 (16.7%) in the hyperamylasemia group, and 11(8.3%) in the no hyperamylasemia group, but the difference was not significant. The length of ICU or emergency critical unit stay and length of hospital stay between the two groups were similar. Among patients who underwent CT evaluation, baseline characteristics stratified by the presence or absence of pancreatitis-like CT findings are shown in Table S1.

**Table 1:** Baseline patient characteristics.

Variable	All patients (n = 169)	Hyperamylasemia (+) (n = 36)	Hyperamylasemia (-) (n = 133)	*p* value
Age, years (IQR)	73.3 (65–85)	75.5 (64–84)	75.0 (65–85)	0.76
Sex, male, n (%)	89 (53)	18 (52)	71 (53)	0.85
Cause of accidental hypothermia, n (%)				
Infection	3(1.7)	0(0)	3 (2.7)	
Substance intoxication	4(2.7)	0(0)	4(3.0)	
Endocrine disease	10(5.9)	2(5.6)	8(6.0)	
Trauma	2(1.1)	0(0)	2(1.5)	
Water immersion	14(8.2)	3(8.3)	11(8.3)	
Other	21(12.4)	6(16.7)	15(11.3)	
Unknown	115(68.0)	25(69.4)	90(67.7)	
Comorbidities, n (%)				
Diabetes mellitus	10(6.0)	0(0)	10(7.5)	
History of pancreatitis	1(0.6)	1(2.8)	0(0)	
Hepatobiliary disease	14(8.2)	6(16.7)	8(6.0)	
Cardiovascular disease	20(11.8)	5(13.9)	15(11.3)	
Dementia	19(11.2)	2(5.6)	17(12.8)	
psychiatric disorders	39(23.0)	7(19.4)	32(24.0)	
Other	73(43.1)	19(52.8)	54(40.6)	
Initial core body temperature, °C (IQR)	28.9 (27.1–30.7)	28.6 (26.3–30.0)	28.9 (27.4–31.0)	0.1
Peak of serum amylase, IU/L (IQR)	138 (70–335)	714 (551–1091)	95 (63–183)	< 0.0001
CT findings suggestive of acute pancreatitis among patients who underwent CT, n(%)	14 (8.2)	13 (36.1)	1 (0.6)	< 0.0001
Administration of catecholamine, n (%)	59 (34.9)	13 (36.1)	46 (34.6)	0.86
Ventilation, n (%)	32 (19.0)	9 (25.0)	23 (17.3)	0.34
Renal replacement therapy, n (%)	4 (2.4)	2 (5.6)	2 (1.5)	0.18
Length of ICU stay, day (IQR)	5 (3–9)	6 (3–9)	5 (3–9)	0.84
Length of hospital stay, day (IQR)	13 (3–29.5)	14 (3–32.8)	13 (3–29.5)	0.84
ICU mortality, n (%)	17 (10.0)	6 (16.7)	11 (8.3)	0.2
Discharge destination, n (%)				
Home	58 (34.3)	14 (38.9)	44 (33.1)	
Transfer to another hospital	88 (52.0)	15 (41.7)	73 (54.9)	
Death	23 (13.6)	7 (19.4)	16 (12.0)	

Baseline characteristics, clinical features, and outcomes of patients with accidental hypothermia stratified by the presence or absence of hyperamylasemia.

IQR: Interquartile range, ICU: intensive care unit

**Fig 2:**
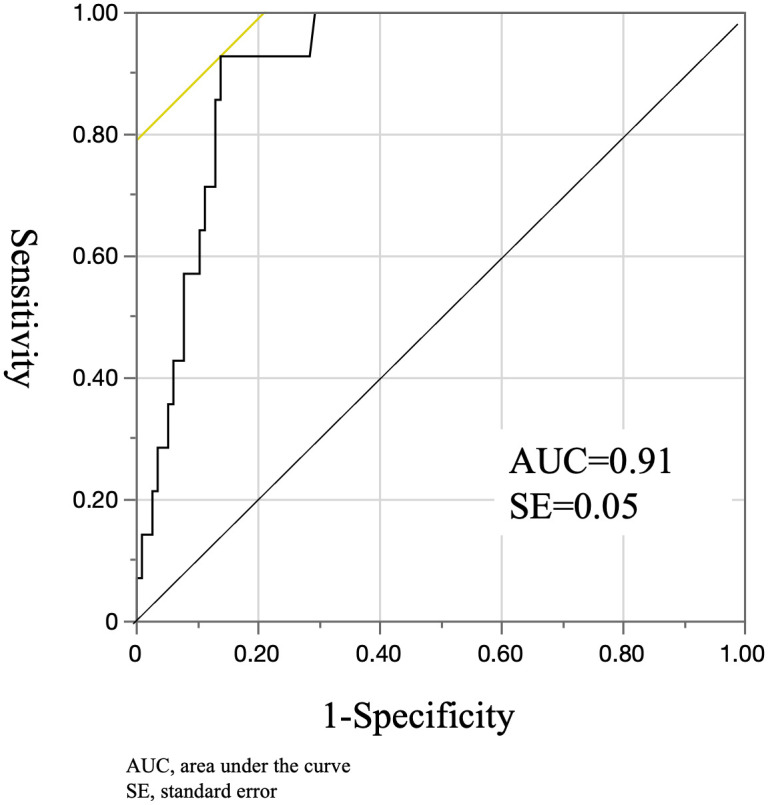
Receiver operating characteristic (ROC) curve of serum amylase for pancreatitis-like CT findings in accidental hypothermia. ROC curve of serum amylase for computed tomography (CT) findings suggestive of pancreatitis in patients with accidental hypothermia. The area under the curve (AUC) was 0.91.

ROC analysis identified 428 IU/L as the optimal cutoff value for Pancreatitis-like CT findings (AUC = 0.91, sensitivity = 0.93, specificity = 0.86) (Fig 2). The positive predictive value (PPV) and negative predictive value (NPV) were 44.8% and 99.0%, respectively.

Table 2 summarizes the clinical characteristics of 13 patients with hyperamylasemia and pancreatitis-like CT findings. Although several patients presented with severe acidemia or required catecholamines and organ support, only two patients required renal replacement therapy and no patients required pancreatic intervention. One patient died on the day of admission. Detailed CT findings, management, and outcomes are summarized in Table 3. Most CT abnormalities consisted of localized peripancreatic fat stranding, fluid collection, or pancreatic enlargement. No patients developed pancreatic necrosis or pancreatitis-related complications requiring invasive treatment. Most patients were managed conservatively with fluids and nutritional support. Rewarming management consisted primarily of warmed intravenous fluids and external rewarming. Short-term outcomes were generally favorable.

**Table 2:** Case series of patients with accidental hypothermia who had hyperamylasemia and CT findings suggestive of acute pancreatitis.

No	Age, years	Sex	Initial core body temperature, °C	Peak serum amylase, IU/L	Use of Catecholamine	pH	Organ support
1	59	Male	29.3	812	None	7.23	Ventilation
2	91	Male	24.0	1172	NA	7.25	None
3	86	Female	26.3	610	None	7.23	None
4	64	Male	34.3	428	None	–	None
5	56	Female	27.1	857	NA	7.05	None
6	77	Female	27.1	5567	NA	7.25	None
7	72	Male	31.1	1029	DA	7.34	None
8	68	Male	29.3	1481	NA	6.88	None
9	86	Female	28.7	733	None	–	None
10	74	Male	27.5	454	None	7.28	None
11	81	Female	25.2	715	NA	7.27	None
12	72	Female	30.6	478	NA	7.07	RRT
13	72	Female	29.7	2563	None	7.09	RRT

Clinical characteristics, management, and outcomes of patients with accidental hypothermia, hyperamylasemia, and pancreatitis-like CT findings. Most patients received conservative treatment, and no invasive procedures were performed.

NA: noradrenaline, DA: dopamine, CT: computed tomography

RRT: renal replacement therapy

**Table 3:** Detailed CT findings, management, and outcomes of patients with hyperamylasemia and CT findings suggestive of acute pancreatitis.

No	CT findings	History of Hepatopancreatobiliary disease	Pancreatitis-related complications	Consultation with gastroenterologists	ICU stay, days	Hospital stay ,days	Discharge	Treatment
1	Peripancreatic fat stranding and fluid collection	None	None	+	29	47	Home	Fluids
2	Localized peripancreatic fat stranding, Pancreatic enlargement	None	None	–	1	1	death	None
3	Localized peripancreatic fat stranding,	History of cholecystectomy	None	+	3	10	Home	Fluids
4	Pancreatic duct dilatation	None	None	+	4	18	Home	Fluids
5	Localized peripancreatic fat stranding,	Cholelithiasis, Pancreatitis	None	+	5	39	Transfer to another hospital	Fluids
6	Peripancreatic fat stranding and fluid collection extending to the anterior pararenal space	None	None	+	6	29	Transfer to another hospital	Fluids
7	Peripancreatic fat stranding and fluid collection extending to the anterior pararenal space	None	None	+	8	36	Home	Fluids
8	Pancreatic enlargement	None	None	+	6	98	Home	Fluids
9	Localized peripancreatic fat stranding, Pancreatic enlargement		None	+	4	24	Transfer to another hospital	Fluids
10	Localized peripancreatic fat stranding and fluid collection	None	None	+	4	9	Transfer to another hospital	Fluids
11	Localized peripancreatic fat stranding and fluid collection	None	None	–	18	18	Transfer to another hospital	None
12	Pancreatic enlargement Localized peripancreatic fluid collection	Cholelithiasis	None	–	9	17	Transfer to another hospital	None
13	Peripancreatic fluid collection, Pancreatic enlargement	None	None	+	8	153	Home	Fluids

Computed tomography (CT) findings, hepatopancreatobiliary history, clinical course, and outcomes of patients with accidental hypothermia who showed pancreatitis-like imaging findings. Most patients had mild imaging abnormalities without pancreatitis-related complications and were managed conservatively with fluid therapy. In Case 4, isolated pancreatic duct dilatation was included as a pancreatitis-like CT finding because gastroenterologists were consulted and the finding was considered clinically relevant to pancreatic involvement.

## Discussion

In this study, we identified 428 IU/L as a serum amylase threshold associated with pancreatitis-like CT findings in patients with accidental hypothermia. This value was close to the level corresponding to three times the institutional upper limit of normal for serum amylase, which is consistent with the enzyme criterion used in the revised Atlanta classification [2]. However, because serum lipase data was unavailable and abdominal symptoms could not be consistently assessed, the serum amylase threshold cannot be interpreted as a diagnostic cutoff for acute pancreatitis. Rather, it may support consideration of CT evaluation in selected patients with accidental hypothermia and hyperamylasemia. Although sensitivity and negative predictive value were high, the positive predictive value was modest. These findings suggest that hyperamylasemia alone is insufficient to diagnose pancreatitis-like imaging findings in accidental hypothermia. Notably, our results demonstrate that pancreatitis-like CT findings were uncommon when serum amylase levels were below 428 IU/L.

Most imaging abnormalities consisted of localized peripancreatic inflammatory changes, and no patients developed pancreatic necrosis or required invasive pancreatic intervention. Most patients were managed conservatively with fluids and nutritional support. Although one patient died on the day of admission and several patients were transferred to other hospitals, pancreatitis-related necrosis or complications requiring invasive pancreatic treatment were not documented in this cohort. As this was a retrospective study, it was not possible to clarify all of the data corresponding to the severity of the pancreatitis in patients with pancreatitis-like imaging findings. However, in patients who received treatment for pancreatitis, the treatment was performed only with fluid administration, and they were discharged from our hospital, with no record of deaths. In previous studies, it was concluded that more than 60% of non-survivors of accidental hypothermia died after more than 2 days because of complications [7,8]. G.-J. van der Ploeg et al. reported that patients who survived for more than 24 h after admission had complications such as pulmonary edema, pneumonia, renal failure, sepsis, and coagulation abnormalities. Although pancreatitis was not included in this survey, multiple organ failure has been pointed out as a cause of death [8].　Previous pathological studies have suggested that pancreatic changes associated with hypothermia may not necessarily correspond to severe clinical pancreatitis [9]. The fact that pancreatic disease alone is not considered to be a direct risk factor for death, and it does not determine the severity of the condition, supports the results of this study.

Previous reports have suggested that the factors involved in prolonging the length of hospital stay in cases of accidental hypothermia are not the presence of complications such as pancreatitis, but rather frailty, indoor conditions, alcoholism, pH, serum potassium levels, and DIC scores [7]. Our study suggested that high amylase levels were not related to prolonged hospitalization.

Treatment for acute pancreatitis in the early phase is fluid resuscitation, which can reduce the risk of organ failure and death [10]. After that, if there is no nutritional intolerance, early enteral nutrition should be considered [11]. The treatment of early acute pancreatitis is centered on fluid and nutritional therapy, and invasive intervention for pancreatic necrosis is recommended after 4 weeks of onset [2]. There were no cases of invasive treatment in this survey, and there was only one case of death. It is possible that there are few severe cases of acute pancreatitis that occur in association with accidental hypothermia, and that conservative management may have contributed to favorable short-term outcomes. Therefore, when hyperamylasemia above 428 IU/L is observed in accidental hypothermia, CT evaluation may help identify pancreatitis-like findings.

It has been reported that patients with accidental hypothermia and acute pancreatitis had significantly lower initial core body temperatures than those without [6].

However, this study found no significant difference in initial core body temperature and hyperamylasemia, and found little relevance to the onset of acute pancreatitis. The severity of accidental hypothermia and death are influenced by a variety of complex factors. They are affected by geographical factors, patient attributes [12,13], living environment [14], medical system [3], and individual educational level [15]. There is a possibility that the results differ between other regions in Japan and our hospital. Therefore, it is desirable to investigate the relationship between initial core body temperature and pancreatitis findings in hospitals in regions with different environmental temperatures.

This study has some limitations. First, this was a single-center retrospective observational study conducted in a regional area of Japan, which may limit the generalizability of the findings and preclude causal inference. Residual confounding cannot be excluded because the severity and outcomes of accidental hypothermia may be influenced by multiple clinical, environmental, and prehospital factors. In addition, the relatively small number of patients with pancreatitis-like CT findings limited the statistical robustness of the analyses and prevented reliable multivariable adjustment. Second, a formal diagnosis and severity assessment of acute pancreatitis could not be consistently performed. Because of the retrospective design, abdominal pain and other clinical findings required for the diagnosis of acute pancreatitis could not be reliably assessed from the medical records. Serum lipase was not routinely available for rapid measurement during the study period, limiting direct comparison between amylase and lipase. CT examinations were generally performed during the initial emergency department evaluation rather than after 48 h from symptom onset, and some CT scans were performed without contrast enhancement in severely ill patients; therefore, standardized radiological severity assessments such as the computed tomography severity index could not be consistently evaluated retrospectively. Moreover, because CT was not performed in all patients, the diagnostic accuracy estimates should be interpreted as test performance among patients who underwent CT evaluation rather than as fully intention-to-diagnose estimates for the entire accidental hypothermia cohort. Thus, the CT abnormalities in this study should be interpreted as pancreatitis-like imaging findings rather than definitive radiological staging of acute pancreatitis.

Despite these limitations, this study provides detailed clinical, radiological, and short-term outcome data on pancreatitis-like findings associated with accidental hypothermia, a condition for which available evidence remains limited. In particular, the case-series analysis suggested that pancreatitis-like imaging findings in this cohort were not associated with pancreatic necrosis or invasive pancreatic intervention, while also highlighting the need for careful evaluation in selected patients.

## Conclusion

In the present study, most patients with pancreatitis-like CT findings showed favorable short-term outcomes and were managed conservatively without invasive pancreatic intervention. These findings suggest that pancreatitis associated with accidental hypothermia may differ from typical severe acute pancreatitis. However, because the pathophysiology and clinical significance remain insufficiently understood and severe cases have been reported previously, careful evaluation and early recognition remain important in clinical practice. Serum amylase levels ≥428 IU/L may support consideration of CT evaluation in selected patients.

## Supporting information

S1 Table Clinical characteristics of patients who underwent CT imaging, stratified by findings suggestive of acute pancreatitis. Clinical characteristics and outcomes of patients with accidental hypothermia who underwent CT imaging, stratified according to the presence or absence of CT findings suggestive of acute pancreatitis. One patient without hyperamylasemia showed CT findings suggestive of acute pancreatitis.
(DOCX)

S2 Table Case series of accidental hypothermia and hyperamylasemia without findings suggestive of acute pancreatitis on CT scans. Clinical characteristics and outcomes of patients with accidental hypothermia and hyperamylasemia without CT evidence of acute pancreatitis. The clinical status at hospital arrival is shown for deceased patients and was recorded as the presenting condition rather than the cause of death. NA: noradrenaline, DA: dopamine, DB:dobutamine, CT: computed tomography.
(DOCX)
