# Supplementary material for: Clinical relevance of hyperamylasemia and pancreatitis-like imaging linked with accidental hypothermia
Source: PLoS One. 2026 Jul 7;21(7):e0353128. doi: 10.1371/journal.pone.0353128 (PMC13340815; doi:10.1371/journal.pone.0353128)
Supplement: S1 Table — Clinical characteristics and outcomes of patients with accidental hypothermia who underwent CT imaging, stratified according to the presence or absence of CT findings suggestive of acute pancreatitis. One patient without hyperamylasemia showed CT findings suggestive of acute pancreatitis. (DOCX) [file pone.0353128.s001.docx]

**Table S1: Clinical characteristics of patients who underwent CT imaging, stratified by findings suggestive of acute pancreatitis**

Clinical characteristics and outcomes of patients with accidental hypothermia who underwent CT imaging, stratified according to the presence or absence of CT findings suggestive of acute pancreatitis. One patient without hyperamylasemia showed CT findings suggestive of acute pancreatitis.

IQR: Interquartile range, ICU:, intensive care unit

| Variable | Finding of pancreatitis  on CT scan (+)  (n = 14) | Finding of pancreatitis  on CT scan (-)  (n = 116) | *p* value |
| --- | --- | --- | --- |
| Age, years (IQR) | 72(62.8-82.5) | 77(57.8-85.0) | 0.33 |
| Sex, male, n (%) | 7 (50.0) | 66 (56.7) | 0.77 |
| Cause of accidental hypothermia, n (%)  Infection  Substance intoxication  Endocrine disease  Trauma  Water immersion  Other  Unknown | 0(0)  0(0)  2(14.3)  0(0)  0(0)  0(0)  12(85.7) | 2(1.7)  2(1.7)  5(4.3)  2(1.7)  8(6.9)  18(15.5)  79(68.1) |  |
| Comorbidities, n (%)  　Diabetes mellitus  **History of pancreatitis**  **Hepatobiliary disease**  Cardiovascular disease  Dementia  psychiatric disorders  Other | 0(0)  1(7.1)  3(21.4)  1(7.1)  0(0)  4(28.6)  7(50.0) | 8(7.0)  0(0)  8(7.0)  13(11.4)  14(12.2)  23(20.2)  62(54.3) |  |
| Initial core body temperature, ℃ (IQR) | 28.1(26.5-29.8) | 28.7(27.0-30.5) | 0.40 |
| Peak of serum amylase, IU/l (IQR) | 724(472-1249) | 133(72-258) | <0.001 |
| Administration of catecholamine, n (%) | 7(50.0) | 47(40.8) | 0.57 |
| Ventilation, n (%) | 3(21.4) | 27(23.5) | 1.0 |
| Renal replacement therapy, n (%) | 2(14.9) | 2(1.7) | 0.06 |
| Length of ICU stay, day (IQR) | 6(4.0-9.5) | 6(3.0-11.0) | 0.7 |
| Length of hospital stay, day (IQR) | 24(16.3-42.0) | 15(2.0-31.0) | 0.07 |
| ICU free days, day (IQR) | 21(15-24) | 21(12-24) | 0.91 |
| Death of ICU stay, n (%) | 1(7.1) | 14(12.7) | 1.0 |
| Discharge destination, n (%)  Home  Transfer to another hospital  Death | 7(50%)  6(42.9%)  1(7.1%) | 30(25.9%)  67 (57.8%)  19(16.4%) |  |
