# Supplementary material for: Clinical relevance of hyperamylasemia and pancreatitis-like imaging linked with accidental hypothermia
Source: PLoS One. 2026 Jul 7;21(7):e0353128. doi: 10.1371/journal.pone.0353128 (PMC13340815; doi:10.1371/journal.pone.0353128)
Supplement: S2 Table — Clinical characteristics and outcomes of patients with accidental hypothermia and hyperamylasemia without CT evidence of acute pancreatitis. The clinical status at hospital arrival is shown for deceased patients and was recorded as the presenting condition rather than the cause of death. NA: noradrenaline, DA: dopamine, DB:dobutamine, CT: computed tomography. (DOCX) [file pone.0353128.s002.docx]

**Table S2: Case series of accidental hypothermia and hyperamylasemia without findings suggestive of acute pancreatitis on CT scans**

Clinical characteristics and outcomes of patients with accidental hypothermia and hyperamylasemia without CT evidence of acute pancreatitis. The clinical status at hospital arrival is shown for deceased patients and was recorded as the presenting condition rather than the cause of death.

NA: noradrenaline, DA: dopamine, DB:dobtamine,　CT: computed tomography

| No | Age group | Sex | Initial core body temperature, ℃ | Peak serum amylase level, IU/L | Catecholamine | pH | Organ support | ICU stay, days | Hospital stay, days | Consultation with gastroenterologists | Discharge | Clinical condition of deceased patients upon arrival at the hospital |
| --- | --- | --- | --- | --- | --- | --- | --- | --- | --- | --- | --- | --- |
| 1 | 60-69 | Male | 25.6 | 635 | None | 7.10 | None | 9 | 34 | - | Home |  |
| 2 | 60-69 | Male | 22.2 | 1077 | None | - | None | 11 | 11 | - | Transfer to another hospital |  |
| 3 | 70-79 | Female | 29.7 | 401 | None | 7.36 | None | 3 | 37 | - | Transfer to another hospital |  |
| 4 | ≧90 | Male | 29.6 | 1075 | None | 7.07 | None | 1 | 1 | - | death | Multiple organ failure |
| 5 | 80-89 | Female | 28.1 | 1097 | None | 7.18 | None | 4 | 10 | - | Transfer to another hospital |  |
| 6 | 50-59 | Male | 28.6 | 812 | NA・DB | 6.69 | ECMO,  Ventilation | 39 | 71 | - | Transfer to another hospital |  |
| 7 | 80-89 | Female | 26.7 | 700 | NA・DA | 6.92 | Ventilation | 1 | 1 | - | death | Out-of-hospital cardiac arrest |
| 8 | 60-69 | Male | 25.2 | 607 | DA | 7.47 | Ventilation | 58 | 135 | - | Home |  |
| 9 | 80-89 | Female | 31.5 | 713 | None | 7.18 | Ventilation | 5 | 8 | - | death | Multiple organ failure |
| 10 | 70-79 | Male | 32.7 | 2120 | NA・DA・DB | 7.17 | Ventilation | １ | １ | - | death | Multiple organ failure |
| 11 | 20-29 | Female | 28.1 | 3317 | None | 7.23 | None | 6 | 7 | - | Home |  |
| 12 | 70-71 | Female | 26.6 | 397 | NA | 7.32 | None | 16 | 21 | - | Transfer to another hospital |  |
| 13 | ≧90 | Female | 26.1 | 1012 | None | 6.80 | None | 1 | 1 | - | death | Cerebral infarction |
| 14 | 70-79 | Male | 28.9 | 1594 | None | 7.08 | None | 1 | 2 | - | Home |  |
| 15 | 80-89 | Male | 30.1 | 826 | None | 7.11 | None | 2 | 3 | - | death | Intracerebral hemorrhage |
| 16 | 60-69 | Male | 29.7 | 450 | NA・DB | 6.97 | Ventilation | 8 | 9 | - | Transfer to another hospital |  |
| 17 | 60-69 | Female | 29.4 | 691 | None | 7.31 | None | 4 | 29 |  | Transfer to another hospital |  |
| 18 | 80-89 | Male | 28.7 | 1213 | None | 7.21 | None | 18 | 19 |  | Transfer to another hospital |  |

Clinical characteristics and outcomes of patients with accidental hypothermia and hyperamylasemia without CT evidence of acute pancreatitis. The clinical status at hospital arrival is shown for deceased patients and was recorded as the presenting condition rather than the cause of death.

NA: noradrenaline, DA: dopamine, DB:dobtamine,　CT: computed tomography
